# SANTIA: a Matlab-based open-source toolbox for artifact detection and removal from extracellular neuronal signals

**DOI:** 10.1186/s40708-021-00135-3

**Published:** 2021-07-20

**Authors:** Marcos Fabietti, Mufti Mahmud, Ahmad Lotfi, M. Shamim Kaiser, Alberto Averna, David J. Guggenmos, Randolph J. Nudo, Michela Chiappalone, Jianhui Chen

**Affiliations:** 1 Department of Computer Science, Nottingham Trent University, Clifton Lane, Nottingham, NG11 8NS UK; 2 Medical Technologies Innovation Facility, Nottingham Trent University, Clifton Lane, Nottingham, NG11 8NS UK; 3 Computing and Informatics Research Centre, Nottingham Trent University, Clifton Lane, Nottingham, NG11 8NS UK; 4 Institute of Information Technology, Jahangirnagar University, Savar, Dhaka, 1342 Bangladesh; 5 Department of Health Sciences, University of Milan, Via di Rudinì, 8, 20142 Milan, Italy; 6 Department of Physical Medicine and Rehabilitation, University of Kansas Medical Center, 3901 Rainbow Blvd, Kansas City, 66160 USA; 7 Department of informatics, Bioengineering, Robotics and System Engineering-DIBRIS, University of Genova, Via All’Opera Pia, 13, 16145 Genoa, Italy; 8 Faculty of Information Technology, International WIC Institute, Beijing University of Technology, Beijing, 100124 China; 9 Beijing International Collaboration Base on Brain Informatics and Wisdom Services, Beijing, 100124 China

**Keywords:** Local field potential, Artifacts, Neural networks, Machine learning, Neuronal signals

## Abstract

Neuronal signals generally represent activation of the neuronal networks and give insights into brain functionalities. They are considered as fingerprints of actions and their processing across different structures of the brain. These recordings generate a large volume of data that are susceptible to noise and artifacts. Therefore, the review of these data to ensure high quality by automatically detecting and removing the artifacts is imperative. Toward this aim, this work proposes a custom-developed automatic artifact removal toolbox named, SANTIA (SigMate Advanced: a Novel Tool for Identification of Artifacts in Neuronal Signals). Developed in Matlab, SANTIA is an open-source toolbox that applies neural network-based machine learning techniques to label and train models to detect artifacts from the invasive neuronal signals known as local field potentials.

## Introduction

Neural recordings give insight into the brain’s structures and functions. The recording systems aim to capture the electrical activity of the biological structures; however, these are not isolated systems and activities from other sources are also recorded. Besides, faulty equipment handling, electrical stimulation, or movements of electrodes can cause distortions in the recordings. As part of the recording process, the recordings must be reviewed to identify corrupted segments and address them, as they are detrimental for any posterior analysis. This includes artifact removal (e.g., filtering, template subtraction, or advanced computational techniques) or discarding the segment.

Each neural recording session produces a huge volume of data, especially if it is obtained over a long period of time and the experiment requires repetition. The amount of data gets multiplied by the number of recording sites. The post-experimental reviewing process consisting of annotating long recordings for evoked responses or unusual activities, which may happen in a much smaller time scale (e.g., 0.1 s in an hour), is a tedious and tiresome task. By automating this task, the researcher can focus on the interpretation task for diagnosis or an application. Employing machine learning (ML) algorithms, which have the ability to learn from patterns to predict unseen data, has been successful in the literature. However, a computational background is required to apply them successfully as there are intricacies such as defining hyper-parameters.

Research groups in the neuroscience community have developed and shared toolboxes for analyzing neural recordings [1–3]. Given the wide arrange of neuronal signals, data formats, analysis techniques, and purposes, each one has advocated their efforts into specific elements. Table 1 lists the available open toolboxes and their functions in regard to aiding noise detection and removal in local field potential signals (LFP). An in-depth analysis of these toolboxes is reported in [4]. Hence, the description below will be dedicated to elaborate on the reported toolboxes.

**Table 1:** Open-source toolboxes and noise detection and removal functionalities

	Artifact detection	Digital filtering	Data visual.	Spectral analysis	Stim. art. removal	File oper.	Multiple formats
Brainstorm [5]	$\checkmark$	$\checkmark$	$\checkmark$	$\checkmark$	X	X	$\checkmark$
BSMART [6]	X	X	$\checkmark$	$\checkmark$	X	X	$\checkmark$
Chronux [7]	X	X	$\checkmark$	$\checkmark$	X	X	X
Elephant [8]	X	X	$\checkmark$	$\checkmark$	X	X	$\checkmark$
Fieldtrip [9]	X	$\checkmark$	$\checkmark$	$\checkmark$	X	X	$\checkmark$
Klusters, NeuroScope, NDManager [10]	X	$\checkmark$	$\checkmark$	$\checkmark$	X	$\checkmark$	$\checkmark$
Neo [11]	X	X	$\checkmark$	X	X	$\checkmark$	$\checkmark$
NeuroChaT [12]	X	X	$\checkmark$	$\checkmark$	X	X	$\checkmark$
Spycode [13]	X	$\checkmark$	$\checkmark$	$\checkmark$	X	X	$\checkmark$
SANTIA	$\checkmark$	$\checkmark$	$\checkmark$	$\checkmark$	$\checkmark$	$\checkmark$	$\checkmark$

Data visualization, stimulation artifact removal and file operations (i.e., file splitting, concatenation, column rearranging)

Brainstorm [5] is an open-source application dedicated to neuronal data visualization and processing, with an emphasis on cortical source estimation techniques and their integration with anatomical magnetic resonance imaging data. It offers an intuitive interface, powerful visualization tools, and the structure of its database allows the user to work at a higher level. BSMART [6] is a toolbox intended for spectral analysis of continuous neural time series data recorded simultaneously from multiple sensors. It is composed mainly of tools for auto-regressive model estimation, spectral quantity analysis, and network analysis. All functionality has been integrated into a graphical user interface (GUI) environment designed for easy accessibility.

Chronux [7] is an open-source Matlab software project for the analysis of neural signals via signal specialized modules for spectral analysis, spike sorting, local regression, audio segmentation, and other tasks. Similarly, Elephant [8] is a Python library for the analysis of electrophysiological data, such as LFP or intracellular voltages. It offers a broad range of functions for analyzing multi-scale data of brain dynamics from experiments and brain simulations, such as signal-based analysis, spike-based analysis, and methods combining both signal types.

FieldTrip [9] is an open-source software package developed for the analysis of electrophysiological data. It supports reading data from a large number of different file formats and includes algorithms for data preprocessing, event-related field/response analysis, parametric and non-parametric spectral analysis, forward and inverse source modeling, connectivity analysis, classification, real-time data processing, and statistical inference. Klusters, NeuroScope, and NDManager [10] are a free software suite for neurophysiological data processing and visualization. NeuroScope is an advanced viewer for electrophysiological and behavioral data with limited editing capabilities, Klusters a graphical cluster cutting application for manual and semi-automatic spike sorting, and NDManager an experimental parameter and data processing manager.

Neo [11] is a tool whose purpose is to handle electrophysiological data in multiple formats. Due to its unique property of being able to read or write the data from or to a variety of commonly used file formats, it is included in the list. NeuroChaT [12] is an Python open-source toolbox created to standardized open-source analysis tools available for the analysis of neuronal signals recorded in vivo in the freely behaving animals.

Spycode [13] is a smart tool for multi-channel data processing which possesses a vast compendium of algorithms for extracting information both at a single channel in addition to at the whole network level, and the capability of autonomously repeating the same set of computational operations to multiple recording streams, all without manual intervention.

Out of the aforementioned toolboxes, the only one that allows for artifact detection is Brainstorm. It allows for manual inspection and automatic detection of artifacts, mainly of muscular and movement origin, by filtering the signals in frequency bands (ocular 1.5–15 Hz; for ECG: 10–40 Hz; for muscle noise and some sensor artifacts: 40–240 Hz and subject movement, eye movements, and dental work 1–7 Hz) and classifying the absolute value of signal with a standard deviation threshold. However, artifacts can span a large bandwidth and studies show that they can overlap with those of the neural signals [14]. As an example, the alpha band (8–12 Hz) can have oscillations of high amplitude and be falsely detected as an artifact.

There is one other toolbox that deals with LFP artifact detection. This is SigMate [15–17], a Matlab-based tool that incorporates standard methods to analyze spikes and electroencephalography (EEG) signals, and in-house solutions for LFP analysis. The functionality provided by SigMate include: artifact removal, both fast [18] and slow [14], angular tuning detection [19], noise characterization [20], cortical layer activation order detection, and network decoding [21–24], sorting of single trial LFP [25–28], etc. It deals with slow stimulus artifact removal through an algorithm that subtracts an estimation of the signal by averaging the peaks and valleys detected in it, eliminating the offset. In addition, it allows for visualization of the spectrogram using short-time Fourier transform of the recording to allocate artifactual frequency bands and allow their filtering, among many other analysis functionalities.

To offer a more competitive toolbox, it has been expanded with new functionalities, reported in Table 2. These include state-of-the-art modules for artifact detection, or the analysis of any number of channels unlike SigMate which is limited to 5. Thus, in this paper, we present the SANTIA toolbox (SigMate Advanced: a Novel Tool for Identification of Artifacts in Neuronal Signals), a friendly user interface that aids the offline identification of artifacts process by simplifying the steps to train powerful computational algorithms with the minimum input of the user. For a wider adoption by the community, the toolbox is freely available online at https://github.com/IgnacioFabietti/SANTIAtoolbox.

**Table 2:** Advancements of SANTIA over SigMate

Toolbox	SAD	UNoC	SE	Up	DF	DV	SA	SAR	FO	MF
SigMate [15]	X	X	X	X	$\checkmark$	$\checkmark$	$\checkmark$	$\checkmark$	$\checkmark$	$\checkmark$
SANTIA	$\checkmark$	$\checkmark$	$\checkmark$	$\checkmark$	$\checkmark$	$\checkmark$	$\checkmark$	$\checkmark$	$\checkmark$	$\checkmark$

*SAD* state-of-the-art artifact detection, *UNoC* unlimited number of channels, *SE* supported environment, *Up* updates, *DF* digital filtering, *DV* data visualization, *SA* spectral analysis, *SAR* stimulation artifact removal, *FO* file operations, *MF* multiple formats

**Fig. 1:**
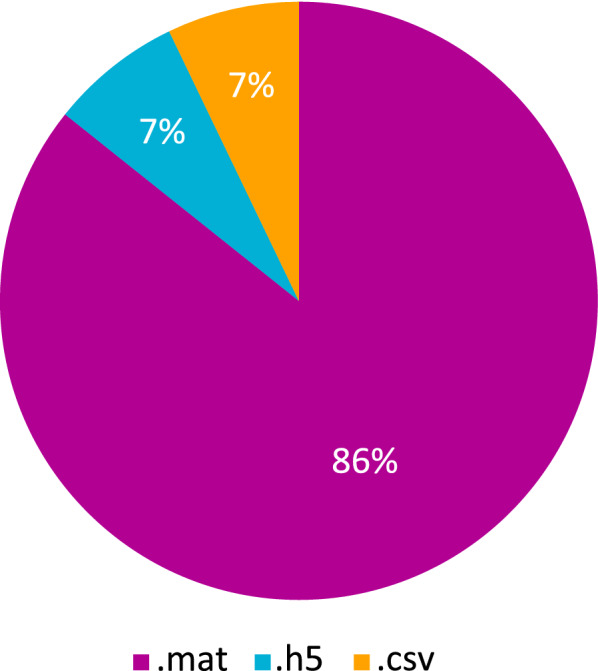
Distribution of formats of local field potential signals in open datasets, extracted from [29]

The recording of neuronal data, especially when using multi-electrode arrays, can lead to electronic files of notable size. Figure 1 illustrates a conducted survey of the formats of invasive neural recordings in open datasets [29]. The data show that ‘.mat’ is the preferred extension for storage by a substantial margin. This emphasizes the necessity to develop tools which address the datasets available in ‘.mat’ format. Therefore, SANTIA was implemented in Matlab and works with single files containing multi-channel data files in a variety of formats. The toolbox only depends on the Deep Learning Toolbox and the basic version of Matlab 2020a and above, therefore can function in any operating system. SANTIA has been developed with the latest app development environment of Matlab, which allows it to be supported for longer and be improved with new modules, such as GUI improvements which are planned for the next update.

The remainder of the paper is composed of 5 sections: Sect. 2 describes the local field potentials; Sect. 3 describes the methods followed by the testing results presented in Sect. 4. Finally, in Sect. 5, discussion and conclusion are presented.

## Local field potentials

**Fig. 2:**
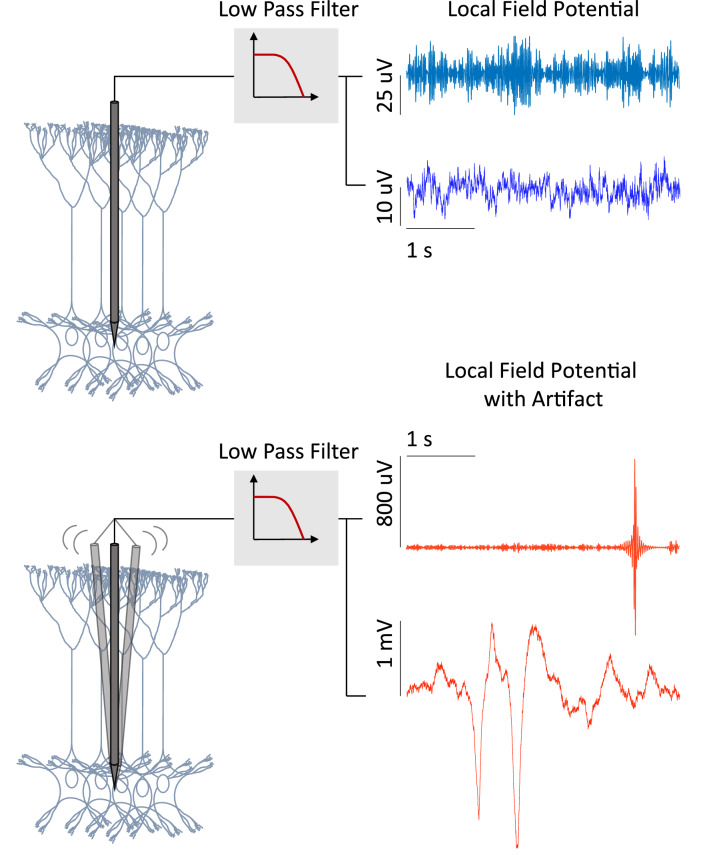
Recording of extracellular neuronal signals from behaving rodents using linear implantable neural probe (shown in gray). Representative local field potential signals with and without movement artifacts are shown from two datasets. The blue traces denote signals without artifacts and the red traces show examples of movement artifacts present in the signals

Local field potentials are invasive neuronal recordings, which are equal to the sum of the activity of a neuronal population, that has been low-pass-filtered under 300 Hz, and whose amplitude ranges from a few micro-volts to hundreds of micro-volts or more depending on the studied structure [30]. They can be recorded by single or multi-channel micro-electrodes (glass micro-pipettes, metal, or silicon electrodes), during in vitro or in vivo experiments to gain insight into the behavior of brain structures, and diagnosis, and are used in application such as brain–machine interfaces. Figure 2 illustrates the concept.

As with all neuronal signals, their recording process can be influenced by internal and external factors, causing artifacts. Within an organism, electric potentials are also generated mainly from ocular, muscle, or heart activity, i.e., electrooculogram, electromyogram, and electrocardiogram, respectively. Examples of external sources include transmission lines, cellphone signals, and faulty experimental setup. Local field potentials in particular can be affected by spike bleed-through [31], light stimulation [32], respiration-coupled oscillations [33], and deep brain stimulation artifacts [34].

The consequences of the presence of artifacts can be detrimental, such as misdiagnosis, disturbance of the study of the brain activity, or causing a brain–machine interface device to be mistakenly operated. Looking at the case of another neuronal signal, EEG signals, the presence of abnormalities raised the median review time from 8.3 to 20.7 min [35]. To make use of these recording successfully, these artifacts must be first identified and then dealt with. The use of computational techniques which are able to learn from complex data patterns has yielded promising results in the field. In the next section, they will be described.

## Methods

### Artifact detection

While there are many contributions on artifact detection in neuronal signals, specially non-invasive ones like EEG, the same cannot be said about LFP. For the latter, the main approach has been the application of ML algorithms in the form of artificial neural networks.

Artificial intelligence has been used for analysis of patterns and classification in diverse fields such as, anomaly detection [29, 36–44], biological data mining [45, 46], disease detection [47–58], monitoring of human [59–62], financial forecasting [63], image analysis [64, 65], and natural language processing [66–68]. Most of the time, these algorithms are composed of multiple layers of neurons for processing of non-linear information and were inspired by how the human brain works. Each neuron calculates an inner product of its inputs ($x_{i}$) and their respective weights ($w_{i}$), and then, the bias (*b*) is added and, finally, the non-linear activation function is applied, which in most cases is a sigmoid function, *tan* hyperbolic, or rectified linear unit. Thus, the output of a neuron ($z_{i}$) can be expressed as detailed in Eq. 1

1 $$z_{i}=f\left(\sum _{i=1}^{n}x_{i}w_{i}+b\right).$$

To propagate the information and train the network, the output of a layer is fed as input to the subsequent unit in the next layer. The result of the final output layer is used as the solution for the problem.

There are many variations of the neural network architecture based on their principles in determining their rules. For example, authors in [69] trained a multi-layered perceptron (MLP) to identify slow-waves in LFP. An MLP is composed of three sections: an input layer, a hidden layer, and an output layer, where the units of the latter two use the non-linear activation defined in Eq. 1. The modeling complex of non-linear relations improves when it contains multiple numbers of hidden layers, compared to a shallow architecture [70].

**Fig. 3:**
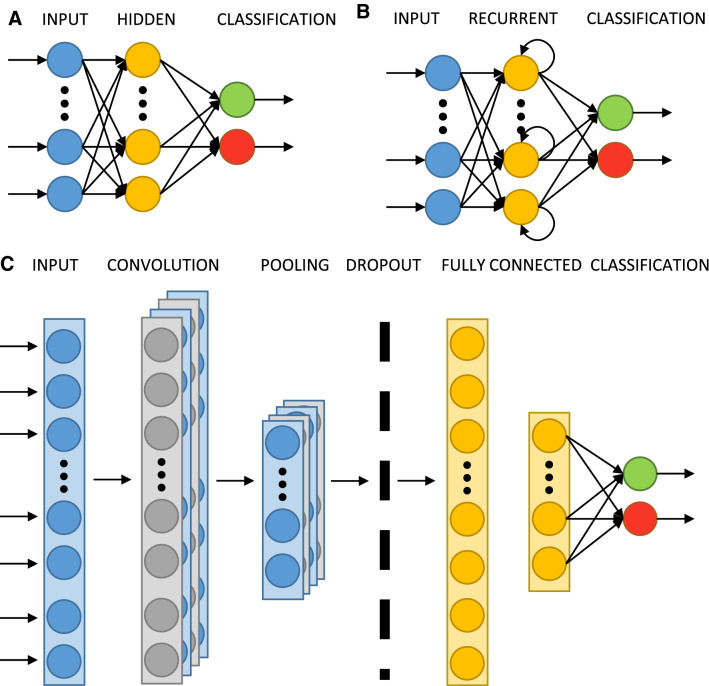
Architectures of different neural network models: multi-layer perceptron (**A**), long short-term memory (**B**), and one-dimension convolutional neural network (**C**). Each circle represents a neuron, multiple rectangles a layer’s depth, and the arrows how the information is propagated throughout each network

In our earlier publications [37], an MLP is employed to identify artifacts in LFP along with two other architectures: long short-term memory (LSTM) networks and one dimensional convolutional neural network (1D-CNN) [71, 72]. The diagrams of the main components of these architectures are depicted in Fig. 3. The LSTM architecture is a type of recurrent network spanning adjacent time steps in a manner that at every point the neurons take the current data input as well as the values of the hidden neurons that collect the information of the previous time steps. On the other hand, convolutional networks are a specific form of neural network that is well suited to computer vision applications due to their capacity to hierarchically abstract representations of spatial operations. A variation of it, designed for problems where the input is a time sequence, is named 1D-CNN.

A comparison of the results obtained can be seen in Table 3. Unlike other machine learning techniques where expertise is required to extract significant features from the signals and which may cause bias in itself, these results indicate that neural networks have the capacity to do it automatically. In addition, it is done in a computationally efficient way: 1-min LFP sampled at 1017 Hz analyzed in 2.27 s equal 26,881 data points analyzed per second. As a negative, the training of the neural network is the step where most time and computational power are consumed.

**Table 3:** Performance comparison, extracted from [72]

Network	Accuracy	Parameters	Computational time (s)
1D-CNN [72]	95.1	561218	2.27 ± 0.13
MLP [37]	93.2	1532	2.57 ± 0.06
LSTM [71]	87.1	4418	3.47 ± 0.04

Having described the classification algorithm that will be used in the toolbox, we proceed to detail its use in the next section.

### Operation

The toolbox can be directly downloaded from the Github repository (https://github.com/IgnacioFabietti/SANTIAtoolbox). Once the toolbox is launched, the GUI provides easy access to all modules. It is important to highlight that SANTIA is a generic environment structured around one single interface in which specific functions were implemented, not a library of functions on top of which a GUI has been added to simplify access.

It is structured in three main modules, designed to perform various processing and analysis on the neuronal signal files. The main functionalities of the first one include: data loading, scaling, reshaping, channel selection, labeling, saving, and 2D display. The second module is composed of: data loading and splitting, hyper-parameter setting, network load or design, network train, test set classification, and threshold setting and saving. Finally, the third one comprehends: data and network loading, classification, and 2D data display and saving.

The GUI allows for user interaction via the selection of functions, parameters, and keyboard inputs, which are processed in the back end. A verification routine executes before running any function to ensure the user has not skipped a step or has not completed the necessary inputs or parameter selection. This minimizes the possible human errors and time expenditure. In case of doubt of the purpose of an element of the GUI, tool tips appear when hovering the cursor over it with a brief explanation.

The functions to display warning messages, generate figures, and compute the labeling, training, or classification are allocated in the back end. These developed features were tested with a dataset recorded from a 4-shank, 16-contact site electrode from anesthetized rats. At the end of each module, the respective outputs can be exported to a ‘.mat’ file, which can easily be utilized in other applications due to the accessibility of the format.

**Fig. 4:**
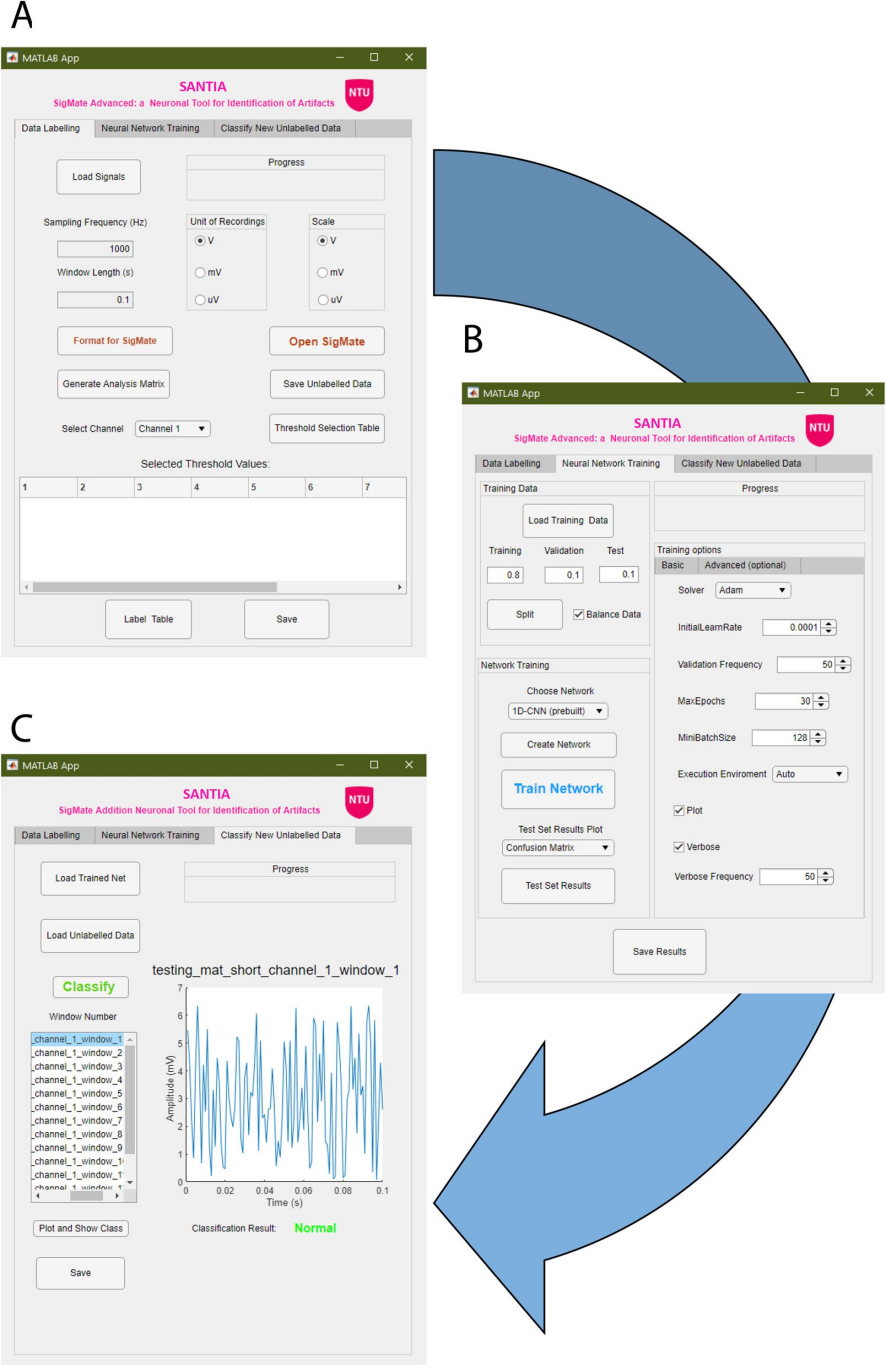
Screenshots of the SANTIA toolbox graphical user interface: Data Labeling (**A**), Neural Network Training (**B**), and Classify New Unlabeled Data (**C**)

**Fig. 5:**
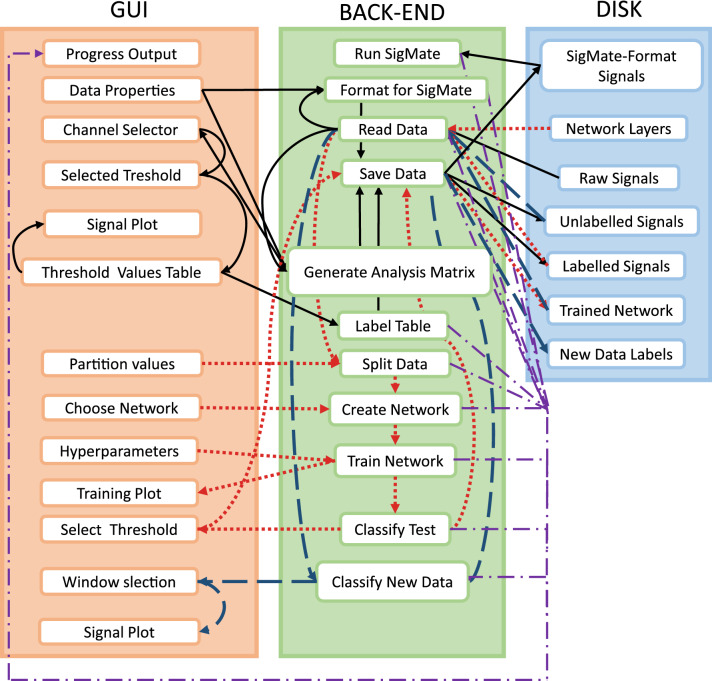
Functional block diagram of the Toolbox.Arrows in black correspond to the “Data Labeling” module , in red to the “Neural Network Training” module, in dark blue to the “Classify New Unlabeled Data” module, and the purple arrows indicate the progress output

**Fig. 6:**
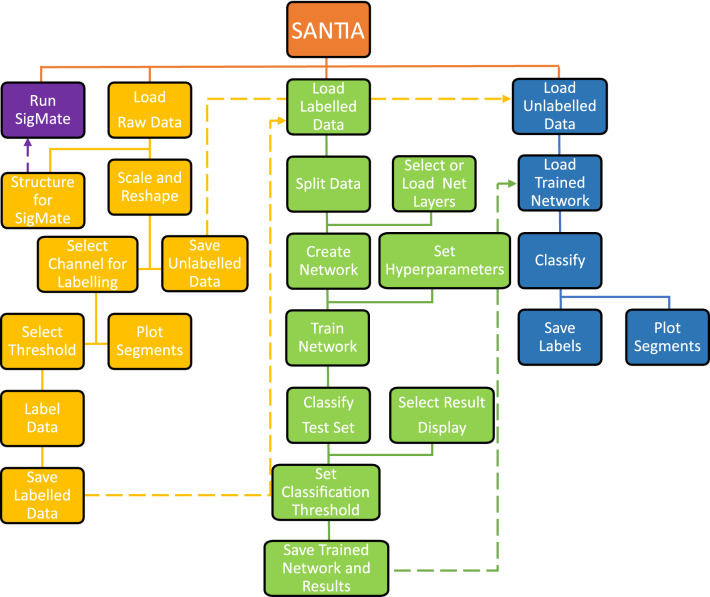
Workflow of the SANTIA toolbox, where the “Data Labeling” modules are colored yellow, the “Neural Network Training” modules in green, and “Classify New Unlabeled Data” modules in blue

The following sections describe the individual modules in greater detail. As a visual aid, Fig. 4 shows the screenshots of the software package, Fig. 5 illustrates the function block diagram, and finally, Fig. 6 shows the workflow diagram.

#### Data labeling

In the first module, the process begins with the ‘Load Signals’ button, which opens the import wizard to load the neural recordings as an $m \times n$ matrix, where m is the number of channels and n are the data points of each channel signal. The compatible formats include ASCII-based text (.txt, .dat, .out, .csv), spreadsheets files (.xls, .xlsx, .xlsm), and Matab files (.set, .mat), which correspond to 93% of the surveyed data in Fig. 1. The user is required to input the sampling frequency in Hz and the window length in seconds that they wish to analyze. In addition, the unit of the recording and the opportunity to scale is presented, as lots of errors happen due to incorrect annotations of magnitudes.

Once all of these parameters have been filled, ‘Generate Analysis Matrix’ will structure the data for posterior analysis. This means that given a window length *w*, and sampling frequency *f*, the $m \times n$ matrix becomes a new $p \times q$ one, where $p=\frac{m \times n}{w \times f}$ and $q= w \times f$. This is incorporated into a table that has row names that follow the format ‘file_id+_channel_+i+_window_j’ where file_id is the name of the LFP data file, *i* the number of channels where $i=1, \ldots, m$ and *j* the corresponding window. In addition, its columns are named: first “window_power” followed by the values of the signal $t_k$ where $k=1, \ldots, q$. As this process involves the creation of *p* amount of row names and window’s power, a memory check is done to read available memory and alert if the usage of more than 80% of the available memory would be needed.

The option to save these data for posterior classification is presented as ‘Save Unlabeled Data’. Otherwise, the user continues by selecting a channel in the drop-down menu or clicking on a table cell and the ‘Threshold Selection Table’ process. This opens a new window with the structured data table, and by clicking on a row, the options to plot the selected window or to define its power as a threshold value appear. As a visual aid, windows with same or higher power are colored red and those with less green, i.e., artifactual and normal, respectively.

In another manner, the user can manually input threshold values in the main app’s table, and once he has completed it for all channels, the data can be labeled and saved as a standardized struct, which contains the original filename, the structured data with its labels, the sampling frequency, window length, the scale, and the threshold values. This information allows researchers to quickly identify different matrices they create and wish to compare. An aid in form of text in the ‘Progress’ banner allows the users to know when each step has been completed, and it is replicated throughout each module.

The user can also structure the data for SigMate analysis. The toolbox expects a $\text{datapoints} (n) \times \text{channels} (m)$ format, with the first column as timestamp and each of the channel’s signal in the following columns. In addition, as it only handles five channels at a time, *m*/5 files have to be generated. Thus, SANTIA transposes the input matrix, generates the timestamp based on the declared sampling frequency, and generates the files. Afterward, it asks the user to select a directory to save them.

#### Neural network training

The second module starts with loading structured data from the previous module. The user is asked to set the values for training, validation, and test splitting. This is common practice to avoid over- and under-fitting results. As artifacts are rare events, the datasets usually present strong imbalance which can cause bias in the training; a tick box for balancing the data is present next to the ‘Split’ button. Clicking it generates three datasets with non-repetitive randomized elements from the original matrix.

This is followed by choosing the network, where the options are MLP, LSTM, 1D-CNN, or for the user to load his/her custom set of layers. This is done by choosing a Matlab file which has a Layer-type variable, i.e., layers that define the architecture of neural networks for deep learning, without the pre-trained weights. These can be modified via console or the Deep Network Designer Toolbox, and for more information, we direct the reader to the mathworks page^1^. While employing different architectures might yield better results, it is also possible that they might not be structured properly and lead to under-fitting, over-fitting, or fail to learn at all. Therefore, a limitation of employing custom networks is the time consumption that takes getting the correct combination of layers, as well as setting parameters such as filter size or activation function. Optionally, the user can customize the training hyper-parameters such as the solver, initial learning rate, and execution environment, among others. These intentionally mirror the ones included in the Deep Network Designer to facilitate its usage to those familiarized with it. These are removed for the MLP option, as it uses a different toolbox (i.e., patternnet of Deep Learning Toolbox [73]), which thus does not allow the same configurations. Clicking the ‘Create Network” button loads the training options and sets the input layer to match the window size.

The ‘Train Network’ button runs the train network function, which inherits the training options and network previously defined. For the 1D-CNN, as the deep learning toolbox is intended for images, the 2D matrices are resized to a 4D vector: 1 × window length × 1  × number of windows, originally intended to be: width × height × channels × number of examples. A display of the training process automatically appears, unless the user decides not to, which enables monitoring the process and early stopping.

Having completed the training, the user can select whether the ‘Classify Test Set’ displays the confusion matrix, the area under the receiver-operating characteristic (AUROC) curve, or opens up a new window where the accuracy, F1 score, and confusion matrix appear along with the possibility to modify the classification threshold (set at 0.5 by default). Finally, ‘Save Results’ creates a struct with data’s filename, the trained network, the training information, the test set’s classification threshold, AUROC, accuracy, F1 score, and confusion matrix.

#### Classify new unlabeled data

The last module begins with loading a trained net along with its classification threshold and unlabeled structured data. After its classification, the options to plot each of the windows with the corresponding color-coded label appear. Finally, users can save the labels as a table with the corresponding window name. Having described the toolbox’s methods, components, and its functions, we proceed to a test case with real recorded LFP.

## Results

In this section, we describe the datasets used to test the app, and the results obtained from them. The artifact detection task carried out by SANTIA toolbox was tested on a daily usage grade Acer TravelMate P278-MGlaptop consisting of 8 gigabyte of RAM and Intel®Core™i7-6500U CPU @ 2.50 GHz processor.

### Dataset 1

A publicly available dataset [74] was used to test the toolbox. Thorough details of the recording and experiment are explained in the article linked to the dataset [75]. Male Long Evans rats (Charles River, Frederick, MD, USA) weighing from 280 to 300 g were trained to walk on a circular treadmill. The recorded LFP were sampled at 2 kHz, and after low-pass filtering, they were amplified times a thousand and band-pass filtered (0.7–150 Hz).

For the purpose of testing the toolbox, only the baseline recordings (prior to ketamine injection) were used. Baseline recordings were composed of at least two 5-min counter-clockwise walking cycles on a slow-moving treadmill and two 40-s rest periods without artifacts. Visual evaluation and videotaped motor activity were used to classify artifact-free periods of 100 s in treadmill-on epochs and 40 to 100 second periods in treadmill-off epochs, which are detailed in Table 4. These labeled artifact-free epochs were used to extract the threshold power value for each channel. It was chosen as the maximum power of the windows in those intervals, for each respective window size.

**Table 4:** Guide to determine best channels and epochs to use of baseline walk and rest recordings in medial prefrontal cortex (mPFC) and the mediodorsal (MD) thalamus, as mentioned in the file named “Coherence Phase Plot Guide”

Rat	mPFC chan1	mPFC chan2	MD chan1	MD chan2	Walk epoch	Rest epoch
KF9	5	6	3	7	960–1160	3780–3820
KF10	3	4	3	8	670–860	1260–1390
KF14	2	6	5	7	740–940	3350–3550
KF15	3	4	5	7	450–640	1600–1700
KF25	2	6	2	5	1480–1680	1700–1800
KF26	1	6	1	6	1180–1380	1050–1150
KF27	2	4	5	8	480–680	2160–2250

The first column is the rat identification, column 2 and 3 the selected two best channels of the mPFC recordings, and 4 and 5 of the MD recordings. Finally, column 6 shows the range of artifact-free epochs during walking and column 7 during resting, respectively [74]

**Fig. 7:**
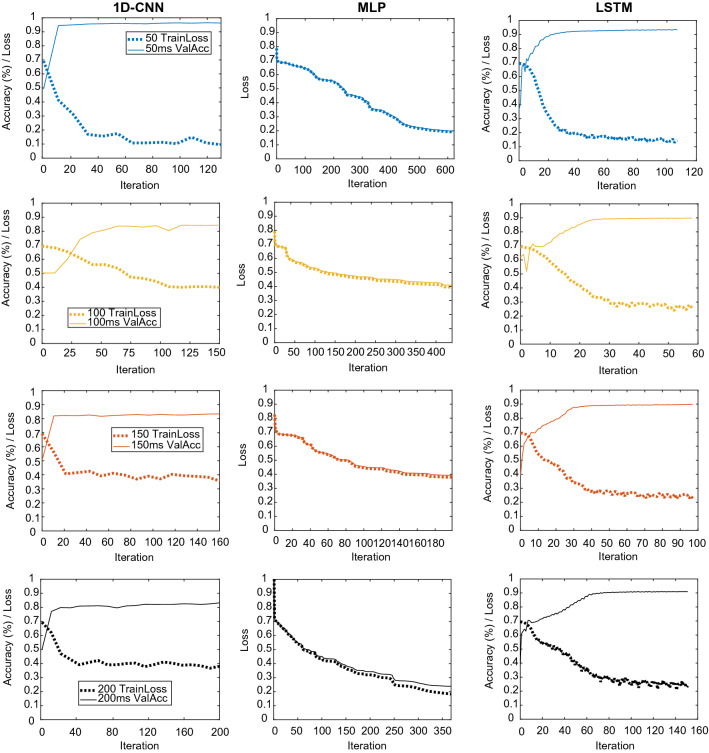
Training plots for models trained with the first dataset

To understand the effect of window size on the artifact detection process, different windows of 0.05, 0.1, 0.15, and 0.2 s were taken and fed to the model. The number of examples obtained after downsampling to balance the classes was on average 275, 687 per window size. For the 1D-CNN and LSTM, the optimization algorithm used was Adam, with an initial learning rate of 0.001, the momentum of 0.9, and a batch size of 1280. On the other hand, the MLP was optimized via a scaled conjugate gradient function. The performance of the models during training is shown in Fig. 7. As they originate from different toolboxes, the MLP does not generate the accuracy throughout the training, and thus, it is not shown.

These results are consistent with previously obtained ones. They indicate that since the filters from the 1D-CNN learn from regions of the signal, instead of the individual values, they are able to learn more robust features of the signals and lead to better classification. Performance on the test sets is similar to that obtained in the validation set, as shown in Table 5. The best test set classification results were achieved by the 50 ms 1D-CNN, an accuracy of $96.5\%$, and an AUROC of 0.993, indicating that the network has been able to learn successfully.

**Table 5:** First dataset’s results for different architectures and sequence length: training loss, validation accuracy, testing accuracy, and testing AUROC

Network	Sequence length (ms)	Training loss	Val. Acc.	Test Acc.	Test AUROC
MLP	50	0.20	0.92	0.92	0.98
	100	0.41	0.82	0.81	0.90
	150	0.39	0.83	0.83	0.90
	200	0.24	0.91	0.91	0.97
1D-CNN	**50**	**0.10**	**0.96**	**0.97**	**0.99**
	100	0.39	0.84	0.84	0.89
	150	0.37	0.83	0.83	0.91
	200	0.36	0.83	0.83	0.91
LSTM	50	0.16	0.93	0.94	0.99
	100	0.26	0.90	0.91	0.97
	150	0.25	0.89	0.90	0.97
	200	0.25	0.91	0.90	0.97

Values pertaining to model’s best performance are highlighted in bold

### Dataset 2

The toolbox was tested using LFP recorded from rats as previously described in [37, 76]. The LFP were downsampled to 1017.3 Hz and low-pass filtered (with a 0–500 Hz cut-off frequency). 294, 592 zero-mean examples were used in this task which were divided into training (80%), validation (10%), and testing (10%) sets, and used to train the models with the same hyper-parameter configuration used with the previous dataset.

**Fig. 8:**
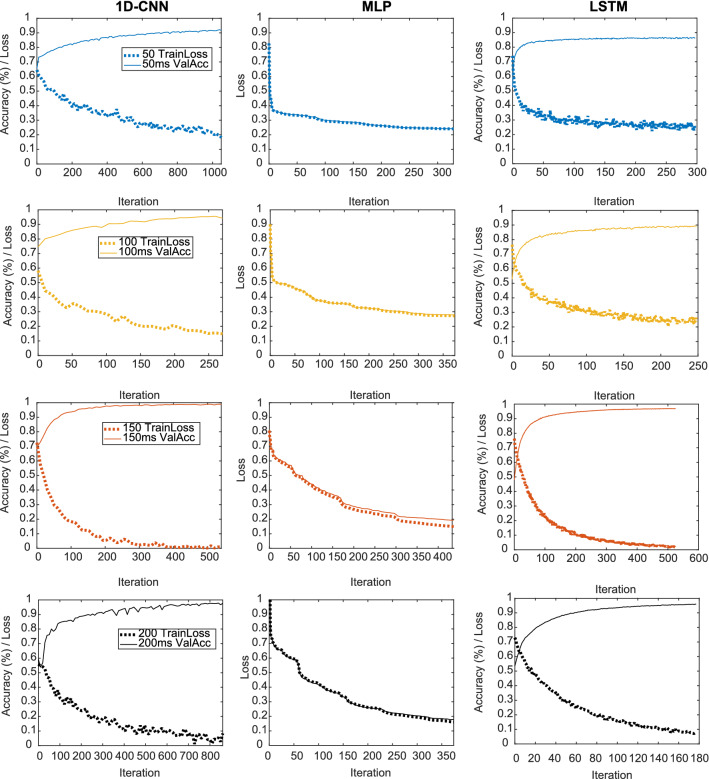
Training plots for models trained with the second dataset

Figure 8 displays the performance of the training and validation set of the different sequence lengths for the two architectures, while the results are compiled in Table 6. Overall, the 1D-CNN outperforms the MLP and LSTM across window sizes. Models with input size of 150 ms have the lowest losses and highest accuracies, meaning that it is the best trade-off between information fed the model and its performance, among the chosen window sizes for this dataset. As shown, different datasets are probable to have different optimal trade-offs between window size and accuracy, due to factors such as sampling rate and artifact frequency.

**Table 6:** Second dataset’s results for different architectures and sequence length: training loss, validation accuracy, testing accuracy, and testing AUROC

Network	Sequence length (ms)	Training loss	Val. Acc.	Test Acc.	Test AUROC
MLP	50	0.24	0.78	0.78	0.857
	100	0.27	0.89	0.86	0.94
	150	0.15	0.94	0.95	0.99
	200	0.16	0.94	0.96	0.98
1D-CNN	50	0.18	0.92	0.91	0.97
	100	0.15	0.94	0.96	0.97
	**150**	**0.01**	**0.99**	**0.99**	**0.99**
	200	0.08	0.98	0.97	0.99
LSTM	50	0.25	0.86	0.86	0.94
	100	0.26	0.89	0.89	0.96
	150	0.02	0.97	0.97	0.99
	200	0.07	0.96	0.96	0.99

Values pertaining to model’s best performance are highlighted in bold

The results are on par with the previous dataset, indicating that the method is robust and possesses generalizability. The 1D-CNN model has shown to obtain the best scores in both cases, establishing it as the better architecture for this type of data.

### Outputs

**Fig. 9:**
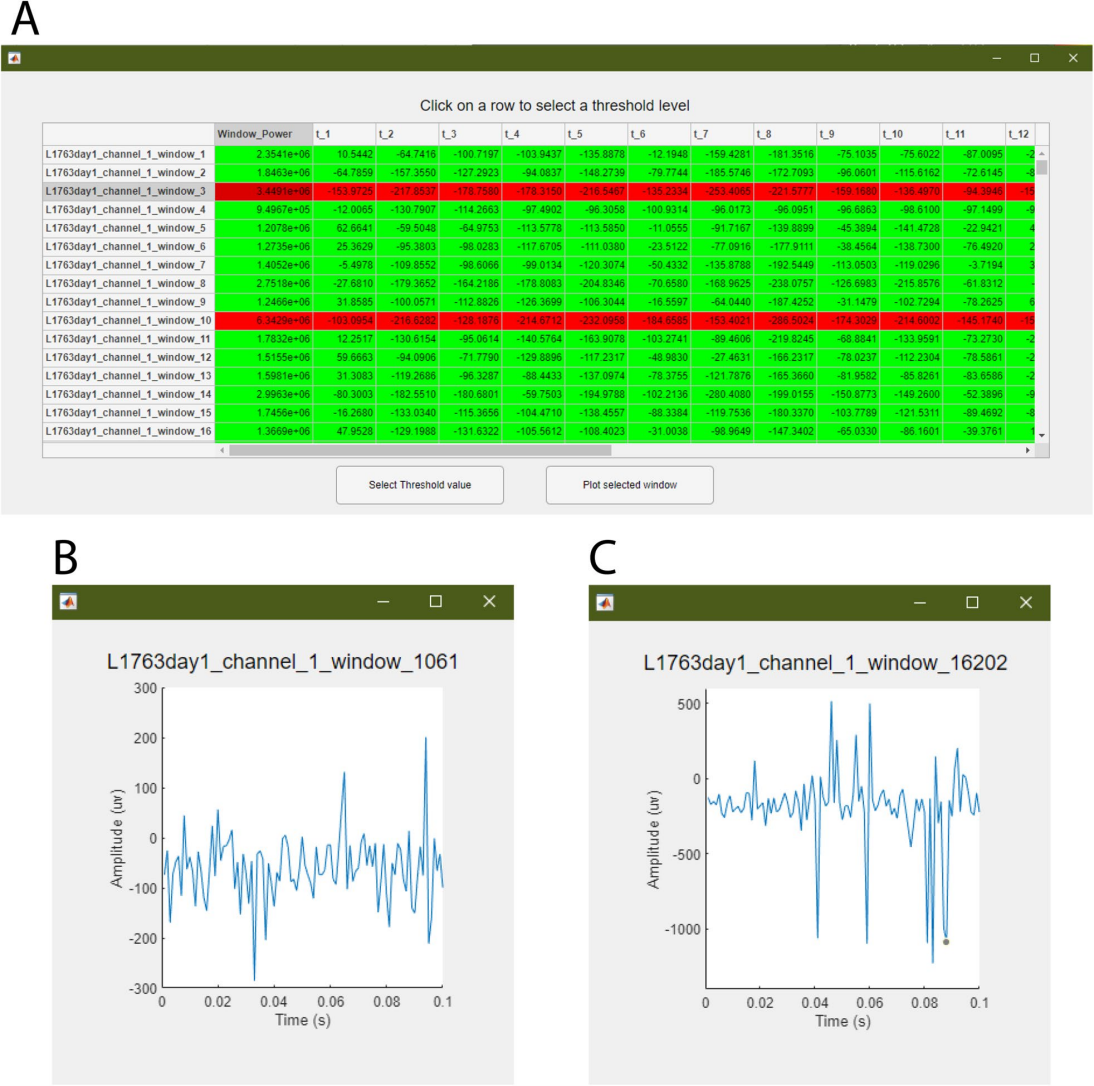
Screenshots of the toolbox’s threshold selection outputs: threshold selection table (**A**), a window of a non-artifactual signal (**B**), and a window of an artifactual signal (**C**)

**Fig. 10:**
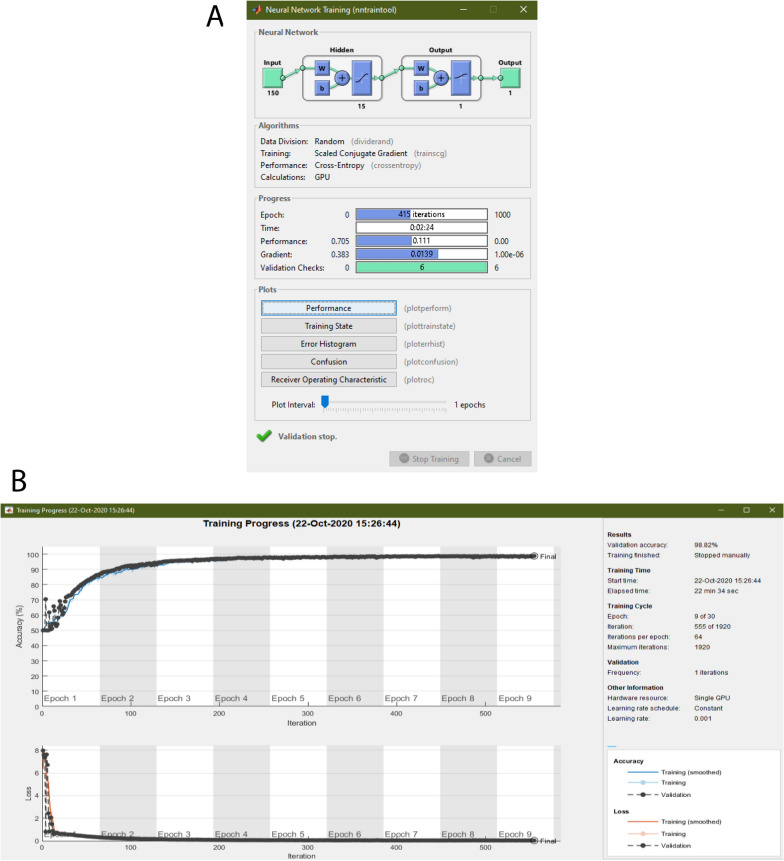
Screenshots of the toolbox’s network training outputs: multi-layer perceptron training process (**A**) and one dimensional convolutional neural network training process (**B**)

**Fig. 11:**
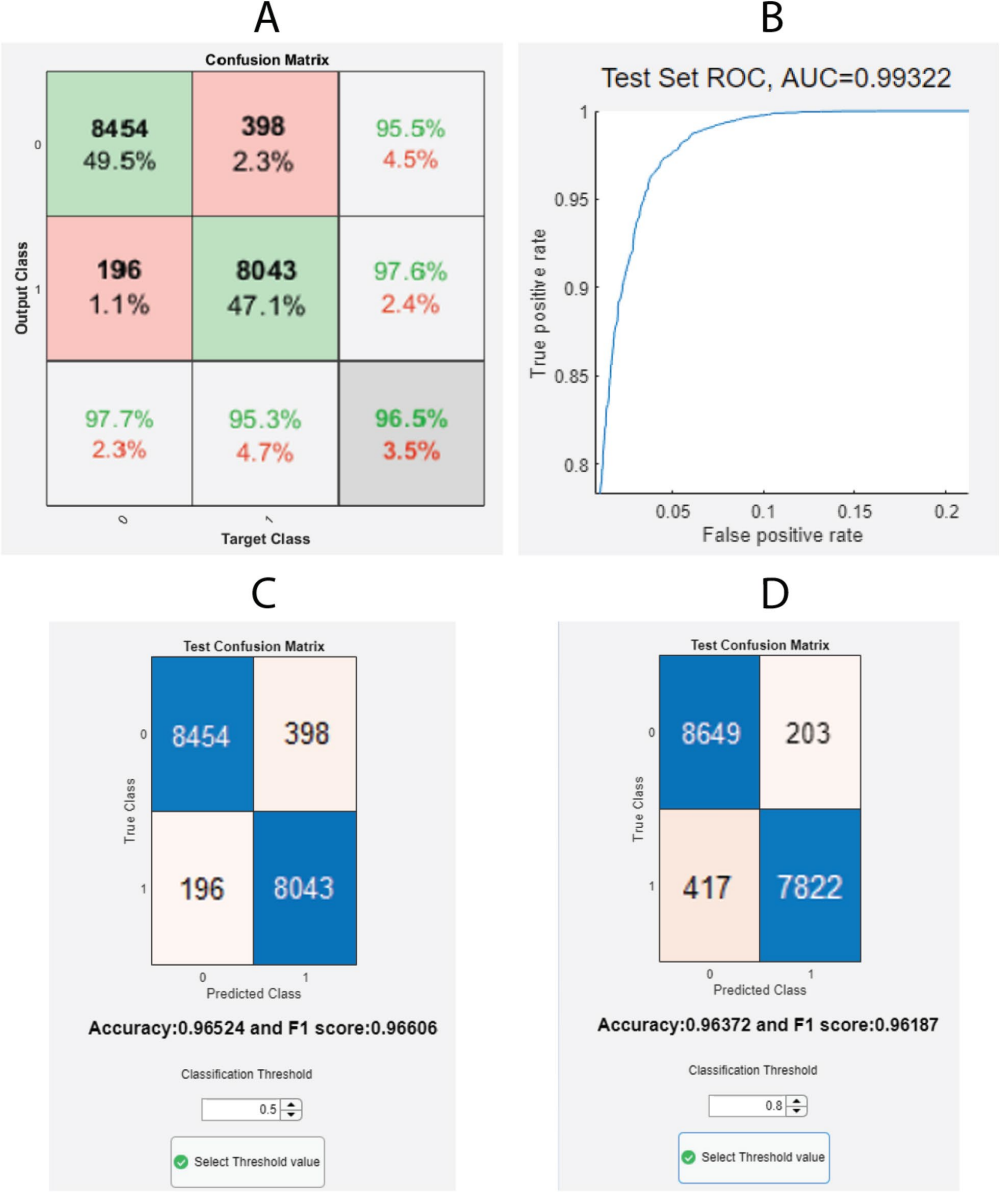
Screenshots of the toolbox’s network test set results outputs: confusion matrix (**A**), AUROC curve (**B**), threshold selection window with default (**C**), and custom values (**D**)

**Fig. 12:**
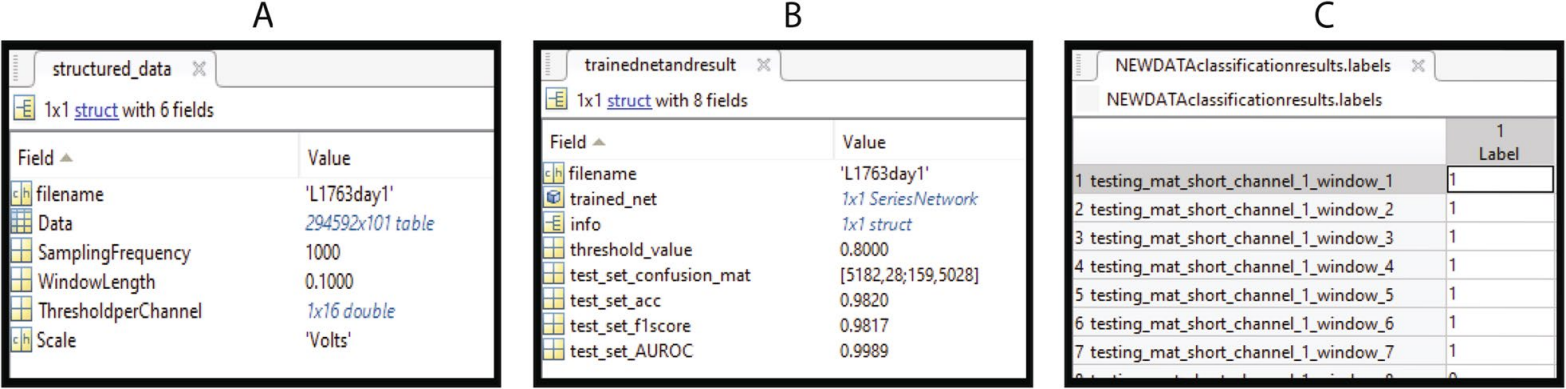
Screenshots of the toolbox’s saved files: labeled data (**A**), trained network and results (**B**), and new data labels (**C**)

Figures 9, 10, 11 and 12 show output windows of the toolbox generated during its operation. Figure 9 displays output windows generated after the data file is loaded. They include the selection of threshold, as shown in Fig. 9A, where green lines show windows representing data above the threshold and red lines show below it, and two representative figures of normal (in Fig. 9B) and artifactual windows (in Fig. 9C). Figure 10 shows the output windows for the neural network training process which currently support MLP, LSTM, and 1D-CNN. As the networks come from different Matlab-toolboxes, their individual configurations require separate processes which are represented in Fig. 10A, B for MLP and 1D-CNN/LSTM, respectively. After having completed the training, the different plots of the test set results of the first dataset for the 50 ms window that were generated are shown in Fig. 11. As a part of allowing the user to evaluate the performance of the models, these figures show the confusion matrix (see Fig. 11A), AUROC curve (see Fig. 11B), and accuracy and F1 score for given classification thresholds (see Fig. 11C, D). Finally, Fig. 12 illustrates the contents of output files generated in each module. These files are saved in Matlab format (.mat) and contain key values for the user to quickly access them, as well as the processed variables needed for any posterior predictions.

## Discussion and conclusion

We developed the SANTIA toolbox to facilitate and standardize the labeling of artifacts in recorded LFP. The simple three-module GUI is designed for researchers without a programming background, and the built-in methodology will allow them to quickly scan and detect the artifacts in their data. It is a project under constant development, and the current version provides an environment where new features can quickly be implemented and adapted to the toolbox. Examples of future developments include:

*Online processing* The tool currently allows for offline labeling, but we wish to expand it a allow the analysis of signals as they are being recorded, to optimize the process.

*Expand format compatibility* There are different libraries for deep learning such as the TensorFlow-Keras, Caffe, and the ONNX (Open Neural Network Exchange) model formats for neural network layers [46]. We wish to add the possibility to read those formats, and in addition the options to import from and save to HDF5 files for the neuronal data under the epHDF standard [77].

*User experience* As this app is adopted by the community, with the feedback, we will improve its shortcomings. The inclusion of testing data, a video tutorial and upgrades of the threshold selection to facilitate its use via graphic elements is also planned. The optimization of some routines via parallelism is also a feature we wish to include, due to the possible large sizes of data files.

*Multi-modality* The incorporation of another source of information (e.g., sensor signal or video) can facilitate and improve the detection of artifacts [78]. A new module would allow the incorporation of such data to facilitate the labeling process or as part of a classification model’s input.

*Artifact removal* Future work will pursue this aspect of artifact analysis as well, with state-of-the-art techniques such as denoising autoencoders [79, 80].

*Portability* As a long-term goal, we consider the implementation in a portable device, e.g., FPGA or Arduino board, to expand the practicality of its usage.

To conclude, SANTIA now represents an option for researchers looking to label artifacts in LFP recordings automatically. This is a work in progress, and some features are yet to be developed; however, the tests with a public and custom dataset have shown promising results. We hope that the neuroscience community adopts this tool, and with their feedback together with our future plans, an improved toolbox is achieved.

## Data Availability

The source-code of the toolbox is available at https://github.com/IgnacioFabietti/SANTIAtoolbox.

## Footnotes

1 https://uk.mathworks.com/help/deeplearning/ref/nnet.cnn.layer.layer.html
